# sRNAdeep: a novel tool for bacterial sRNA prediction based on DistilBERT encoding mode and deep learning algorithms

**DOI:** 10.1186/s12864-024-10951-6

**Published:** 2024-10-31

**Authors:** Weiye Qian, Jiawei Sun, Tianyi Liu, Zhiyuan Yang, Stephen Kwok-Wing Tsui

**Affiliations:** 1 School of Artificial Intelligence, Hangzhou Dianzi University, Hangzhou, 310018 P.R. China; 2 School of Biomedical Sciences, The Chinese University of Hong Kong, Hong Kong SAR, China; 3 Hong Kong Bioinformatics Centre, the Chinese University of Hong Kong, Hong Kong SAR, China

**Keywords:** Bacterial sRNA, Deep learning, Genome analysis, *Mycobacterium tuberculosis*

## Abstract

**Background:**

Bacterial small regulatory RNA (sRNA) plays a crucial role in cell metabolism and could be used as a new potential drug target in the treatment of pathogen-induced disease. However, experimental methods for identifying sRNAs still require a large investment of human and material resources.

**Methods:**

In this study, we propose a novel sRNA prediction model called sRNAdeep based on the DistilBERT feature extraction and TextCNN methods. The sRNA and non-sRNA sequences of bacteria were considered as sentences and then fed into a composite model consisting of deep learning models to evaluate classification performance.

**Results:**

By filtering sRNAs from BSRD database, we obtained a validation dataset comprised of 2438 positive and 4730 negative samples. The benchmark experiments showed that sRNAdeep displayed better performance in the various indexes compared to previous sRNA prediction tools. By applying our tool to *Mycobacterium tuberculosis* (MTB) genome, we have identified 21 sRNAs within the intergenic and intron regions. A set of 272 targeted genes regulated by these sRNAs were also captured in MTB. The coding proteins of two genes (lysX and icd1) are implicated in drug response, with significant active sites related to drug resistance mechanisms of MTB.

**Conclusion:**

In conclusion, our newly developed sRNAdeep can help researchers identify bacterial sRNAs more precisely and can be freely available from https://github.com/pyajagod/sRNAdeep.git.

**Supplementary Information:**

The online version contains supplementary material available at 10.1186/s12864-024-10951-6.

## Introduction

Bacterial small regulatory RNA (sRNA) refers to a class of non-coding RNA with around 50–500 nucleotides in length, which plays an important role in post-transcriptional gene regulation [1]. These sRNAs can regulate gene expression by targeting mRNAs, which can affect mRNA stability, translation, and transcription termination. They can be divided into cis-encoded and trans-encoded sRNAs [2]. Cis-encoded sRNAs are transcribed from the same DNA strand as their target mRNA, while trans-encoded sRNAs are transcribed from a different DNA strand or a distant genomic location from their target mRNA. Some bacterial sRNAs act as regulators of stress responses, virulence factors, and metabolic pathways [3]. Studying bacterial sRNAs has provided insights into the complex regulatory networks that bacteria employ to adapt to changing environments and optimize their survival strategies. Understanding bacterial sRNAs may have implications for the development of novel antimicrobial strategies and biotechnological applications.

With the development of novel biometrics and deep learning algorithms, some techniques for the identification of bacterial sRNAs have emerged. In 2017, Barman et al. developed a support vector machine (SVM) with a *k*-mer encoded strategy to identify sRNAs in *Salmonella Typhimurium* LT2 (SLT2) and achieved an accuracy of 88.35% [4]. In 2019, Eppenhof et al. applied a set of effective features such as predicted secondary structure, open reading frame, and Rho-independent terminator, to identify bacterial small RNAs with machine learning classifiers [5, 6]. In 2021, Kumar et al. developed the tool PresRAT that combined RNA sequence and secondary structure features to identify sRNAs [7]. The identification of sRNA is not satisfied because of two reasons. One reason is due to the low number of known sRNAs in bacteria, the other reason is some defects in converting sRNAs into features using *k*-mer strategy. Therefore, it leads to the traditional machine learning algorithms failing to learn its features effectively, and these algorithms are prone to overfitting, resulting in poor model applicability.

By using Generative Adversarial Networks (GAN) algorithm, the training samples can be well augmented to prevent overfitting. The GAN algorithm was proposed by Goodfellow et al. in 2014, which utilizes the confrontation between the generator and the discriminator to achieve the best fit [8]. The main advantage of GAN is in generating data but not in model prediction. Therefore, researchers often apply the GAN algorithm together with other deep learning algorithms, for example, Convolutional Neural Network (CNN), in biological problems [9]. Tan et al. used the combination of GAN and CNN algorithms to augment the ultrasound images of myositis, and then test the model in another independent dataset, achieving a high prediction accuracy of 92.23% [10].

In bioinformatics field, the *k*-mer feature is widely used to encode sequences with different lengths into a vector with fixed dimensions. However, the dimensionality of the generated feature vector increases rapidly when the *k*-value becomes large, which results in a very sparse vector [11]. To address the limitations of *k*-mer strategy, the BERT (Bidirectional Encoder Representations from Transformers) model in natural language processing (NLP) field was applied to extract features of sRNA sequences [12]. BERT model takes the position and order into account when encoding features. The DistilBERT is based on BERT with knowledge distillation and parametric quantity compression, which enables us to obtain results approximating BERT with limited resources [13].

In this study, we introduced a new sRNA prediction model based on the DistilBERT and TextCNN. We have compared our sRNAdeep with previous existing tools (PresRAT and sRNARanking) in the benchmark dataset and tested our performance in an independent dataset. To further verify the effectiveness of our tool, we applied sRNAdeep in *Mycobacterium tuberculosis* (MTB), a common drug-resistant bacterium with a high frequency of genomic mutation [14]. These experimental results showed that our newly developed tool achieved satisfactory improvements in bacterial sRNA identification and explanation of drug-resistant mechanism in MTB.

## Results

### Data description

**Fig. 1:**
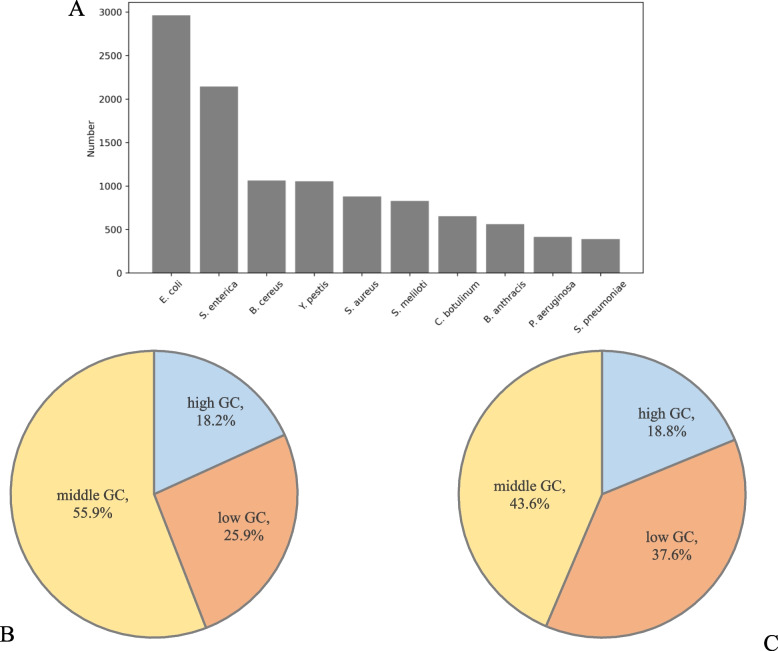
The statistical information of sRNA in benchmark dataset. **A** Histogram of the sRNA distribution of the top ten bacteria. **B** The percentage of different GC proportions of sRNAs in training set. **C** The percentage of different GC proportions of sRNAs in testing set

In this study, we retrieved bacterial sRNAs from BSRD database and collected 30,581 sequences with experimental evidence. These sequences originated from 1033 bacteria with different features. The distribution of sRNA number is illustrated in Fig. 1A. We found that the sRNA number of *E. coli* is the highest in current database. *E. coli* is a model bacterium in scientific research, thus its sRNAs were more easily found. The sRNA number of other bacteria, such as *S. enterica*, *B. cereus,* and *Y. pestis* is also very high.

In addition, a set of 29,566 protein-coding RNAs of common bacteria were obtained from NCBI and applied as negative samples. After filtering by criteria mentioned in “Material and methods” paragraph, the training set consisted of 9754 positive and 18,922 negative samples, while the testing set consisted of 2813 positive and 1239 negative samples (Table 1). Subsequently, we have also calculated the GC proportion of each RNA sequence. In training set, 18.2%, 55.9%, and 25.9% sequences were classified into the groups of high, middle, and low GC proportion, respectively (Fig. 1B). In testing set, 18.8%, 43.6%, and 37.6% sequences were classified into the groups of high, middle, and low GC proportion, respectively (Fig. 1C).

**Table 1:** Statistical information of sample numbers in benchmark dataset

Dataset	Positive sample	Negative sample
Training set	9754	18922
Validation set	2438	4730
Testing set	2813	1239

### The best length of *k*-mer

**Fig. 2:**
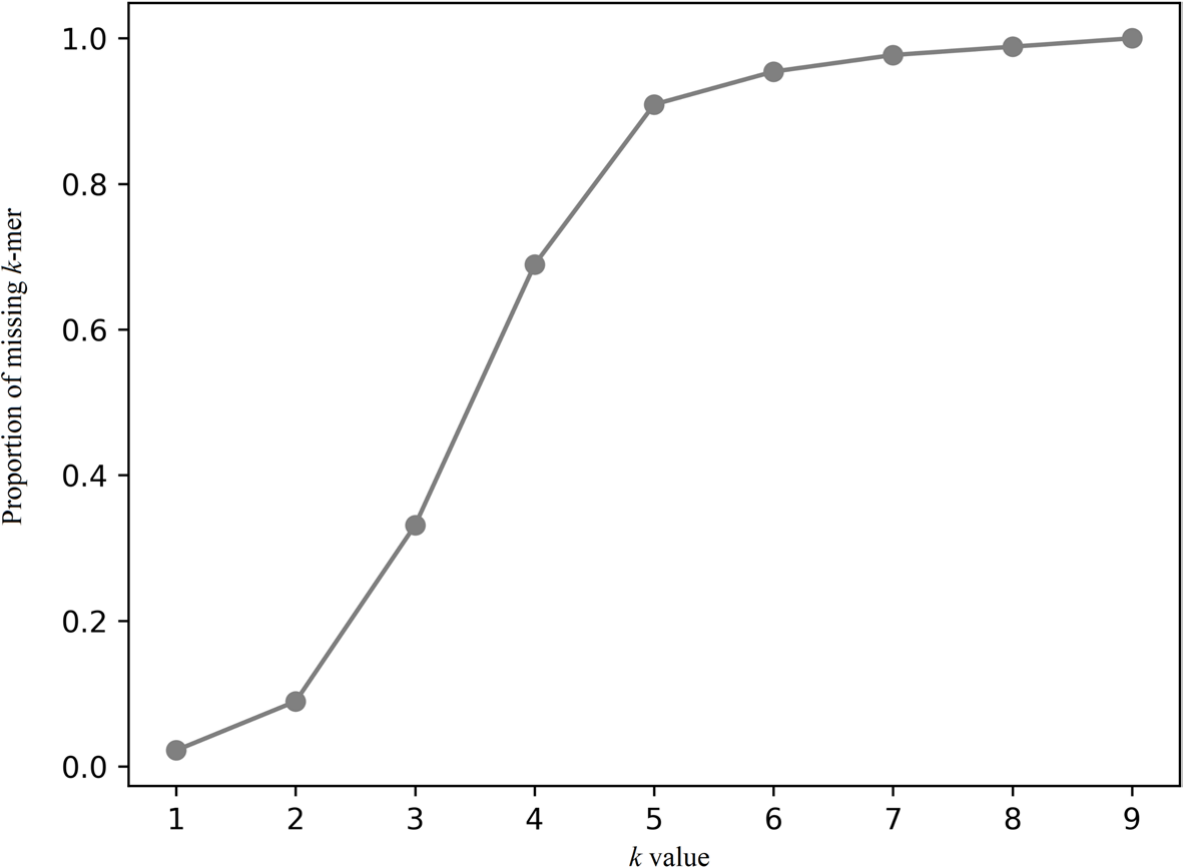
The proportion of missing *k*-mer in different values of *k*. If a k-mer was absent in the sequences, we denoted it as “missing k-mer”

We used machine learning models to test the performance in different lengths of *k*-mer. Using a lower or higher *k* will result in underfitting or overfitting, respectively. When *k* is taken as 1, each *k*-mer contains only one base and lacks contextual information, preventing the model from learning enough information and leading to underfitting. Similarly, when *k* is greater than 4, a proportion of more than 80% of k-mers are missing in feature extraction process (Fig. 2). It leads to data sparsity, which may cause the model to rely too much on specific features, leading to overfitting.

Subsequently, we applied three machine learning models (logistic regression, random forest, support vector machine) to test the performance under different *k*-mer strategies (2-mer, 3-mer, 4-mer) and the results were shown in Table 2. RF has the best performance under all values of *k*. When *k* is taken as 2, although the SPE of both LR and SVM can reach 0.8683 and 0.8835, respectively, the value of SEN is only 0.5075 and 0.4782. which is much smaller compared to the values of *k* equal to 3 and 4. The SEN value of RF is also much smaller than the value of SPE, which indicates that the identification of sRNA has a poor performance when *k* is equal to 2. When *k* is equal to 3, all the metrics of the model are much higher than the other two situations. Combining the results in Fig. 2, when* k* is equal to 3, the data sparsity is much smaller than that when *k* is 4. Although the model with k = 4 exhibits superior performance compared to that with k = 3, the sparse 4-mer count may result in severe overfitting [15]. This suggests that it is more reasonable to take *k* as 3.

**Table 2:** Performance of different machine learning models with different lengths of *k*-mer strategy. LR: logistic regression; RF: random forest; SVM: support vector machine; ACC: Accuracy; SEN: Sensitivity; SPE: Specificity; MCC: Matthew’s correlation coefficient; PRE: Precision; FSC: F1-score; AUC: Area Under the Curve

Index	2-mer			3-mer			4-mer		
	LR	RF	SVM	LR	RF	SVM	LR	RF	SVM
ACC	0.746	0. 9004	0.746	0.838	0.940	0.798	0.893	0.946	0.804
SEN	0.508	0. 8061	0.478	0.725	0.888	0.575	0.819	0.900	0.539
SPE	0.868	0. 9489	0.884	0.896	0.967	0.913	0.931	0.970	0.940
MCC	0.406	0. 7749	0.401	0.633	0.865	0.532	0.759	0.880	0.546
PRE	0.665	0. 8905	0.679	0.782	0.933	0.773	0.859	0.940	0.823
FSC	0.575	0. 8462	0.561	0.752	0.910	0.659	0.839	0.920	0.652
AUC	0.688	0. 8775	0.681	0.810	0.927	0.744	0.875	0.935	0.740

### Three sequence-encoded methods

In above paragraph, we have classified the data into training, validation, and testing sets. The validation set was comprised of 2438 positive and 4730 negative samples. To select the suitable method for encoding sRNAs into numerical vectors, we compared the performance of three sequence-encoded methods (*k*-mer, TF-IDF, DistilBERT) on the validation set. The performance of 5-fold cross-validation was reported in Table 3. It was shown that the performance of DistilBERT method is distantly better than those of the other two methods (*k*-mer and TF-IDF). All seven performance indexes of DistilBERT method are higher than 0.9, especially for AUC and SPE, which reached 0.9620 and 0.9738, respectively. In contrast, the ACC values of the other two methods ( *k*-mer and TF-IDF) were significantly low, which were both less than 0.7. By DistilBERT method, we can reduce the misidentification rate while maintaining the accuracy rate in identifying sRNAs. Thus, we applied DistilBERT methods in the subsequent study.

**Table 3:** Performance of three sequence-encoded methods on sRNA. The abbreviations are the same as those in Table 2

Index	*k*-mer	TF-IDF	DistilBERT
ACC	0.6562	0.6490	0.9659
SEN	0.7044	0.6813	0.9501
SPE	0.5627	0.5863	0.9738
MCC	0.2599	0.2577	0.9238
PRE	0.7576	0.7616	0.9489
FSC	0.7300	0.7192	0.9494
AUC	0.6335	0.6337	0.9620

### The performance of TextCNN algorithm

To test the performance of different strategies, we have compared the results of TextCNN, and TextCNN-GAN based on DistilBERT method. The detailed performances of comparative experiments are listed in Table S1. The results show that the proposed TextCNN-GAN strategy has more significant performance in five metrics: ACC, SEN, MCC, FSC, and AUC, compared to the only TextCNN strategy (Table 4). In particular, the average ACC and SEN of TextCNN-GAN strategy were 2.5% and 4.28% higher than those of TextCNN, respectively.

**Table 4:** Performance of sRNA prediction by TextCNN-GAN and TextCNN strategy

Index	Strategy	1st-Fold	2nd-Fold	3rd-Fold	4th-Fold	5th-Fold	Mean ± SD
ACC	TextCNN-GAN	0.9706	0.9667	0.9538	0.9674	0.9595	0.9636 ± 0.0001
	TextCNN	0.9571	0.9548	0.9344	0.9654	0.8813	0.9386 ± 0.0046
SEN	TextCNN-GAN	0.9772	0.9652	0.9438	0.9854	0.9563	0.9656 ± 0.0011
	TextCNN	0.9513	0.9467	0.9122	0.9691	0.8347	0.9228 ± 0.0114
FSC	TextCNN-GAN	0.9788	0.9757	0.9660	0.9767	0.9704	0.9735 ± 0.0114
	TextCNN	0.9685	0.9668	0.9507	0.9750	0.9071	0.9536 ± 0.0148
MCC	TextCNN-GAN	0.9310	0.9232	0.8968	0.9228	0.9074	0.9162 ± 0.0001
	TextCNN	0.9025	0.8983	0.8598	0.9194	0.7680	0.8696 ± 0.0019
AUC	TextCNN-GAN	0.9664	0.9676	0.9602	0.9560	0.9616	0.9624 ± 0.0008
	TextCNN	0.9607	0.9600	0.9484	0.9631	0.9109	0.9486 ± 0.0195

There are two reasons for the performance improvement of TextCNN-GAN strategy. One reason is that the GAN algorithm incorporates features of TextCNN that allow it to efficiently capture localized features in sRNA data. The other reason is that GAN algorithm enables the analysis of the features of sRNA sequences and generates new effective data based on these features, increasing the generalization ability of the model. The combination of these two factors improves the prediction performance of TextCNN-GAN strategy. On the other hand, the non-sRNAs are very diverse and different, so it is difficult for GAN to generate sufficiently diverse samples, which will have an effect on the generalization ability of the model, resulting in higher SPE and PRE values for a TextCNN strategy than for TextCNN-GAN strategy. Based on these analyses, we selected TextCNN-GAN strategy in our sRNAdeep model.

### Comparing results with other prediction tools

**Fig. 3:**
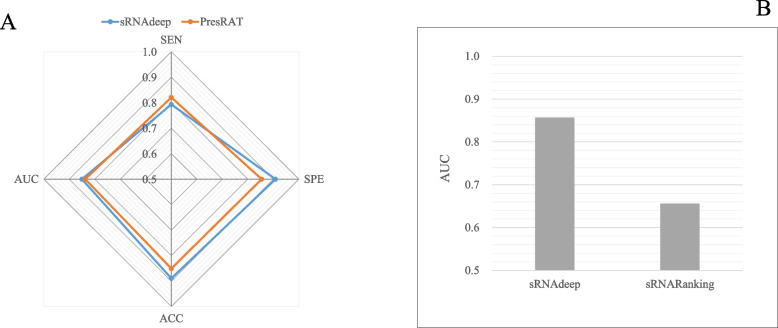
Comparative experimental results of sRNAdeep and PresRAT, sRNARanking. **A** Comparative analysis of sRNAdeep and PresRAT; **B** Comparative analysis of sRNAdeep and sRNARanking

To demonstrate the validity of our model, sRNAdeep was used to compare with two previously published tools: PresRAT and sRNARanking. The PresRAT determines whether an RNA is sRNA by its secondary structure information in the collected sRNA dataset. The sRNARanking applied multiple features, such as open reading frame and promoter site, to predict putative sRNAs in a dataset called STL2. Because the best parameters of these two tools were not provided in the publication, thus we tested the performance of our sRNAdeep in their datasets, respectively. The results of comparative experiments in their datasets are shown in Fig. 3. It was indicated that sRNAdeep has better performance than PresRAT in sRNA identification of its datasets in three indexes (Fig. 3A). The sRNAdeep got a satisfied SPE value of 0.9082, while PresRAT got an SPE value of 0.8541. The ACC value of sRNAdeep was 0.8894 and the ACC value of PresRAT was 0.8508. The SEN of sRNAdeep is slightly smaller than PresRAT, but the running time of sRNAdeep is much smaller than that of PresRAT. By comparing the performance in STL2 dataset, our sRNAdeep got a satisfied AUC value of 0.8571 and sRNARanking got a relatively low AUC value of 0.656 (Fig. 3B). These results further indicated that our tool is more helpful in discovering sRNAs.

### Identified sRNA in MTB

To identify reliable sRNAs in MTB, the non-coding region of MTB genome was aligned against known sRNA sequences by BLAST with the threshold: alignment identity ≥ 80. Subsequently, the RNA sequences were discriminated by our sRNAdeep, and 21 sRNAs remained (Table 5). The sequences of these sRNAs are shown in the Table S2. Notably, all these sRNAs are less than 200 nucleotides, while four sRNAs are shorter than 50 nucleotides. MFEs of most sRNAs are all less than -20 kcal/mol, indicating their inherent stability. Particularly noteworthy is sRNA-1, which not only exhibits MFEs less than -60 kcal/mol but also possesses the smallest average MFEs, suggesting exceptionally strong stability in MTB.

**Table 5:** The details of sRNAs identified in MTB Genomes

Accession	Length	AT	CG	MFE (kcal/mol)
sRNA-1	79	30.4%	69.6%	-62.2
sRNA-2	102	29.4%	70.6%	-58.5
sRNA-3	107	36.5%	63.6%	-56.6
sRNA-4	89	31.5%	68.5%	-46
sRNA-5	120	28.3%	71.7%	-53.6
sRNA-6	103	35.9%	63.1%	-45.3
sRNA-7	156	35.9%	64.1%	-65.7
sRNA-8	63	31.8%	68.3%	-26.2
sRNA-9	72	30.6%	69.4%	-29.4
sRNA-10	100	30.0%	70.0%	-37.8
sRNA-11	109	33.9%	66.1%	-41.1
sRNA-12	93	37.6%	62.4%	-34
sRNA-13	140	37.1%	62.9%	-50.7
sRNA-14	103	33.0%	66.0%	-34.8
sRNA-15	108	41.7%	58.3%	-36.7
sRNA-16	68	35.3%	64.7%	-21.7
sRNA-17	71	29.6%	70.4%	-21.4
sRNA-18	46	34.8%	65.2%	-13.5
sRNA-19	45	46.7%	53.3%	-11.9
sRNA-20	74	40.5%	59.5%	-16.3
sRNA-21	34	47.1%	52.9%	-4.9

### Target genes regulated by sRNAs

**Fig. 4:**
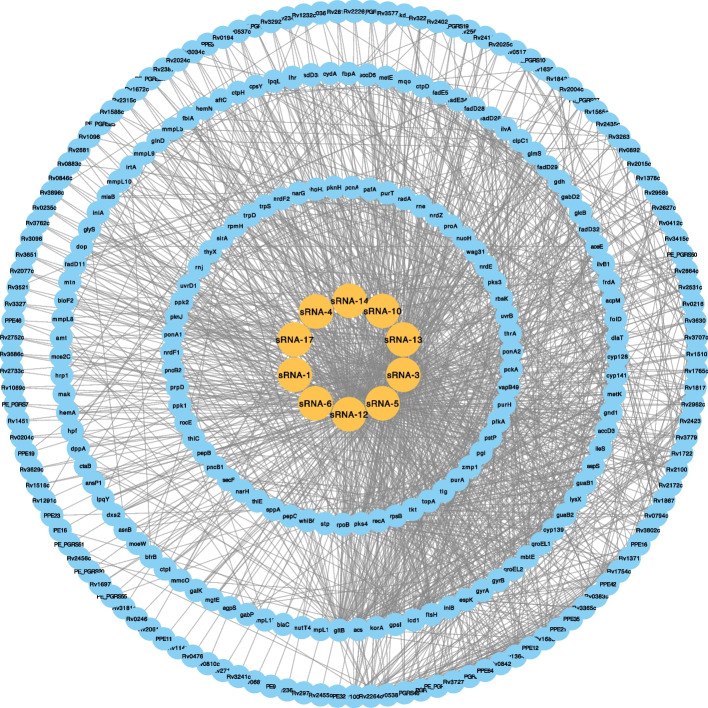
The sRNA-gene regulation network. The sRNAs were shown in yellow nodes and genes were shown in blue nodes

**Fig. 5:**
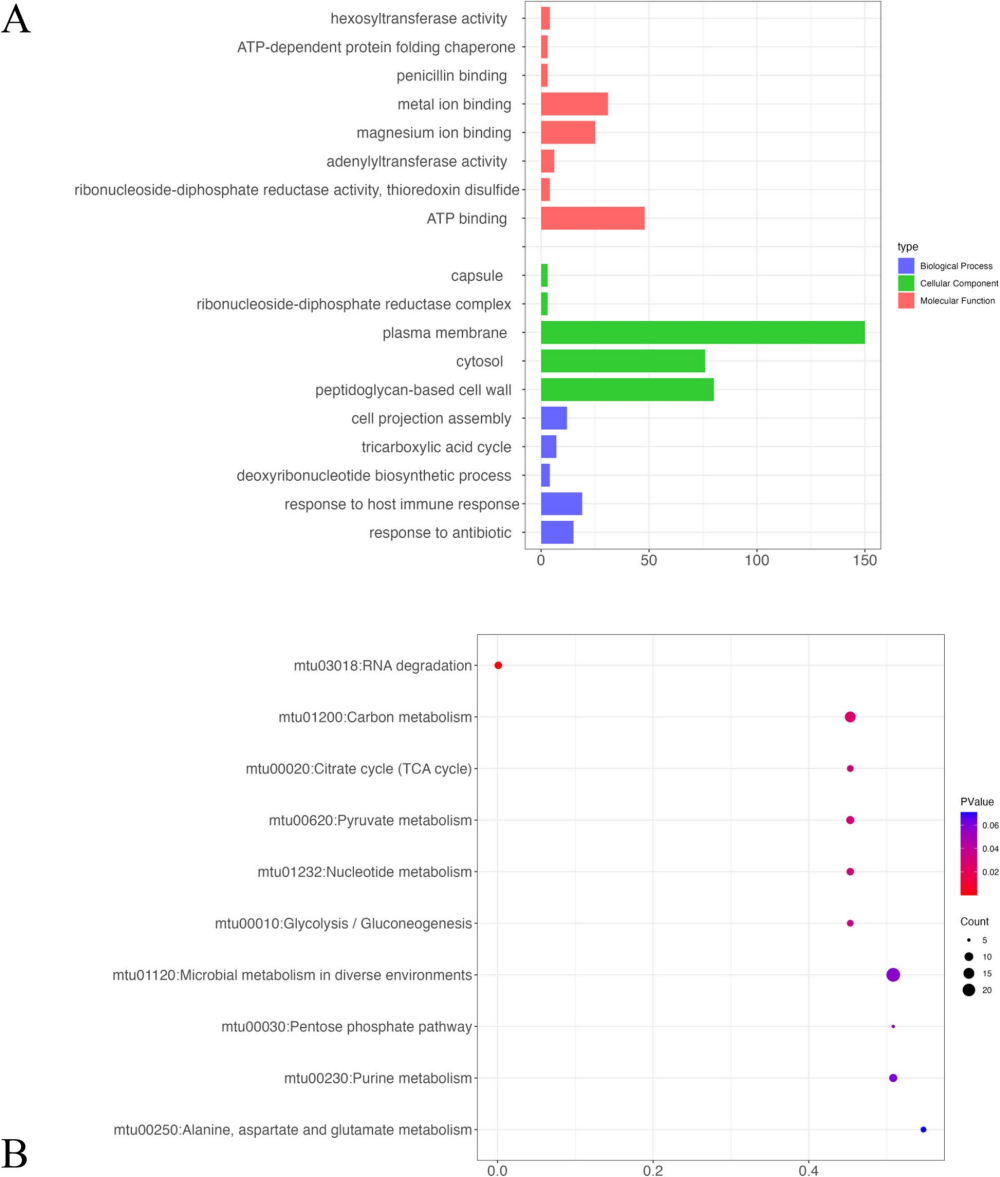
Functional enrichment analysis of sRNA targeted genes. **A** Gene ontology analysis of targeted genes; **B** KEGG pathway enrichment of targeted genes. CC: cellular component; MF: molecular function; BP: biological process

To identify the genes regulated by sRNAs, we calculated the binding possibility of RNAs and known genes in MTB by TargetRNA3. In this study, a total of 272 target genes regulated by sRNAs were screened. The details of all genes are provided in Table S3. The sub-network of protein–protein interaction (PPI) of these 272 genes was retrieved from STRING database. This PPI sub-network and sRNA-regulated-gene sub-network were put together to draw a full sRNA-gene interaction network (Fig. 4). The obtained network is composed of 290 nodes and 1662 edges in this study. Some sRNA could regulate a large number of genes in MTB. For example, four RNAs (sRNA-3, sRNA-5, sRNA-12, and sRNA-13) can regulate more than 100 genes. We further conducted the gene ontology (GO) enrichment analysis of these 272 genes and the result is shown in Fig. 5A. The number of genes related to plasma membrane is much higher than in other categories, with more than 75 members. This result indicated that sRNA intends to regulate membrane proteins, which may be involved in molecule transportation. A set of 15 genes (GYRA, RV2864C, GYRB, PONA2, STP, LYSX, ILES, TIG, BLAC, PONA1, MMPL3, RPOB, RECA, INIA, and RV0194) are related to response to antibiotics. This provides a new perspective on investigating drug resistance mechanism in *Mycobacterium tuberculosis*. The result of KEGG pathway analysis is shown in Fig. 5B. The number of genes related to “Microbial metabolism in diverse environments” is highest in this analysis with an extremely low p-value less than 0.05. In addition, although the number of genes related to “RNA degradation” is small, the *p*-values of pathways are also less than 0.05. These results indicated that our identified sRNA could regulate critical genes in the metabolic pathways of this pathogen.

### Possible active sites in MTB

PrankWeb was used to identify potential binding sites of important proteins encoded by targeted genes. [16]. A set of 7 targeted genes was found to encode proteins with active residue range (Table 6). These genes were regulated by different sRNAs. For example, sRNA-4 was found to regulate gene Rv3138c. The minimum free energy for sRNA-4 and Rv3138c binding was -138.58 kcal/mol with a *p*-value of 2.01E-11 and a binding probability of 0.9052. This indicated that the binding of sRNA-4 and Rv3138c was very stable in the biological process. This protein contained an active residue range (87 ~ 127) for ligand or enzyme binding. The accurate active sites of this protein are 92, 94, and 95. These active sites could be used for drug discovery in the treatment of *Mycobacterium tuberculosis*.

**Table 6:** Information of active sites in coding proteins of targeted genes

Accession	Targeted gene	Energy (kcal/mol)	*p*-value	Probability	Residue Range	Active sites
sRNA-1	Rv2061c	-54.21	2.05E-12	0.955	109–132	109
sRNA-3	lysX	-164.19	1.65E-05	0.906	1093–1131	1093, 1094, 1096
sRNA-4	Rv3181c	-138.58	2.01E-11	0.905	89–127	92,94,95
sRNA-5	icd1	-105.24	8.36E-04	0.763	245–282	258,261,262,280
sRNA-6	Rv0810c	-162.47	6.15E-06	0.947	4–42	15,18,19
sRNA-10	thiC	-147.88	6.21E-07	0.760	507–545	/
sRNA-12	PE_PGRS50	-11.43	8.73E-04	0.759	59–70	59,60,63
sRNA-13	PE_PGRS50	-11.82	4.33E-04	0.794	1408–1427	/
sRNA-14	thiE	-176.13	6.79E-08	0.935	155–193	176 ~ 180,183
sRNA-17	gpsI	-105.24	3.60E-13	0.945	570–608	/

## Discussion

Small regulatory RNAs (sRNAs) are short RNA molecules found in bacteria that typically range from 50 to 500 nucleotides in length. They play crucial roles in post-transcriptional gene regulation by binding to target mRNAs, influencing their stability and translation efficiency. Currently, emerging biological and deep learning methodologies have facilitated the detection of bacterial sRNAs. However, existing approaches encounter limitations due to the scarcity of known bacterial sRNAs and challenges in converting sRNAs into effective features [17].

In this study, we retrieved known bacterial sRNAs from BSRD database and collected 30,581 sequences of 1033 bacteria. Before putting raw RNA into the machine learning models, the RNA sequence should be encoded into a numeric vector. In bioinformatics field, the *k*-mer feature is widely used to encode RNA sequences with different lengths into a vector with fixed dimensions. We used machine learning models to test the performance in different lengths of *k*-mer. The *k* in these machine learning models was chosen in 2, 3, or 4. When *k* is equal to 3, all the metrics of the model are much higher than in the other two situations, thus we take *k* as 3 in the sequence-encoded model.

To compare the effectiveness of different models, we have compared three sequence-encoded models (*k*-mer, TF-IDF, DistilBERT) in sRNA identification. Results showed that the performance of DistilBERT method is distantly better than those of other two methods (*k*-mer and TF-IDF). The dimensionality of the generated feature increases rapidly when the *k* value becomes large, which results in a very sparse vector. Our DistilBERT model could distantly capture information that spans the entire RNA sequence, which made our performance better.

In addition, there is a problem that the relatively small number of samples with known sRNAs leads to easy overfitting when using the model to train the samples. GAN is a powerful deep-learning framework designed for generating high-quality data [18]. The GAN algorithms can effectively augment training samples, mitigating overfitting issues. The basic principle of GAN is to improve the quality of generated data through adversarial interactions between the generator and the discriminator. This dynamic process ensures that the generative power of the generator and the discriminative power of the discriminator are continuously improved [19].

The features generated by the DistilBERT model are combined and fed into the TextCNN model. TextCNN is a useful deep-learning algorithm for text classification tasks. In TextCNN model, sRNA can be viewed as a one-dimensional image and a one-dimensional convolutional layer can be used to extract text features. TextCNN uses a one-dimensional convolutional layer and a maximum pooling layer to extract sequence features [20]. The features of TextCNN are applied to perform convolutional operations on the output features of the GAN module.

To test the performance of different strategies, we have compared the results of different deep learning methods based on DistilBERT feature extraction strategy. Results show that TextCNN-GAN strategy is more suitable to ensure the accuracy rate of sRNAs with a reduced false recognition rate. Besides, sRNAdeep was used to compare with two previously published tools (sRNARanking and PresRAT). Our sRNAdeep got a satisfied AUC value of 0.8571, while the AUC value of sRNARanking is 0.656. The ACC value of sRNAdeep was 0.8894, while the ACC value of PresRAT was 0.8508. The performance in the situation of different GC proportions was also tested. We have calculated the proportion of high, medium, and low GC proportions of sRNAs and non-sRNAs in the training and test sets, respectively. Results showed that the performances of sRNA identification in the groups of different GC proportions are similar (Table S4). We have also calculated the specific deviation of predicted sRNA and true sRNA numbers in different GC proportions. We found most of the deviation is very small in three groups with high, medium, and low GC proportions (Figure S1). This result showed our method is better than current existing method.

Subsequently, we applied our tool to identify sRNAs in the MTB genome. MTB could cause tuberculosis, one of the severe health challenges globally. The targeted genes of sRNAs were also predicted, and functional enrichment analyses were conducted. Notably, these genes are significantly enriched in three GO terms: “cytosol”, “peptidoglycan-based cell wall”, and “plasma membrane”. The cytosol is a part of the cytoplasm and refers to the fluid portion of the cytoplasm. Many drugs bind to proteins within the cytoplasm, and small molecules often target cytoplasmic proteins [21, 22]. Additionally, the impact of tetracycline on peptidoglycan-based cell wall proteins showed the complex interplay between cell wall composition and antibiotic resistance, presenting promising opportunities for therapeutic interventions [23]. Besides, the active sites of coding proteins of these genes were predicted by PrankWeb. Among these coding proteins, several proteins exhibit effective active sites, such as lysX and icd1. The active sites of the lysX comprise residues 1093, 1094, and 1096. For the icd1 gene, active sites are identified at residues 258, 261, 262, and 280. In previous studies, mutations in lysX in MTB resulted in increased virulence and altered host–pathogen interactions [24].

Although our sRNAdeep performed well in this study, there are still some limitations of our work. One of the limitations is that the data source is single. The sRNAs were provided by BSRD database and non-sRNAs were retrieved from the coding regions of bacteria. Our method is actually learning to differentiate between protein-coding regions and sRNAs. The other limitation is that the performance of the untrained distilBERT is still not perfect, and a pre-trained large language model needs to be developed specifically for sRNA identification in the future.

## Conclusion

To effectively improve the prediction of bacterial sRNAs, a novel prediction tool called sRNAdeep is proposed in this study. This new tool not only applies the sequence-encoded method DistilBERT but also integrates multiple deep learning methods to construct a composite model for bacterial sRNA identification. After preprocessing the dataset, a composite model consisting of TextCNN and GAN was constructed to learn the unique features obtained from the DistilBERT approach. Based on the output of TextCNN-GAN, a classification module consisting of fully connected layers is employed to identify bacterial sRNA. Benchmark studies showed that sRNAdeep outperformed the other sRNA prediction tools in terms of many indexes. In addition, by applying our tool to MTB genome, we have identified 21 sRNAs within the intergenic and intron regions. A set of 272 targeted genes regulated by these sRNAs were captured. The coding proteins of two genes (lysX and icd1) are found to implicate in drug response of MTB, with significant active sites related to drug resistance mechanisms. In conclusion, our newly developed sRNAdeep can help researchers identify bacterial sRNAs more precisely.

## Materials and methods

### Dataset retrieving

We retrieved known bacterial sRNAs from BSRD [25] and collected 30,581 sequences with experimental evidence. These sequences originate from 1033 bacterial species. A set of 29,566 protein-coding RNAs of common bacteria were obtained from NCBI and applied as negative samples. On the basis of the obtained sRNAs and protein-coding RNAs, we generated the dataset to train and test our model by following steps: (1) Select sRNAs and coding RNAs with lengths ranging from 50 to 500; (2) RNAs similar in testing and training set will be removed by BLAST sequence alignment; (3) 80% of the RNA will be selected as the training set and 20% as the testing set; (4) Encode these RNAs into word vectors with fixed length. 20% of the training set was used as the validation set in the fivefold cross-validation process.

**Fig. 6:**
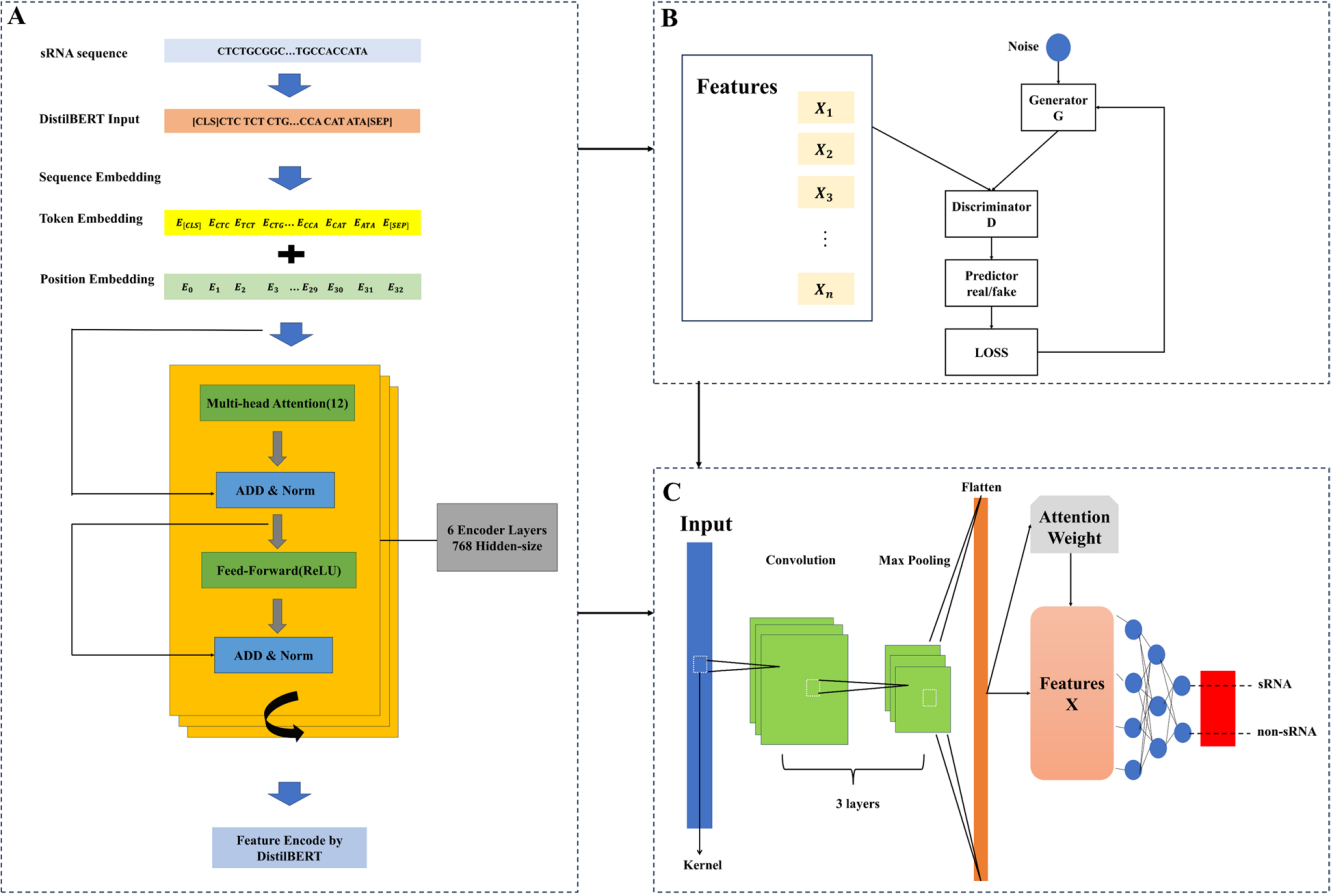
Overall flowchart of our method. **A** Feature Encode by DistilBERT; **B** GAN model; **C** TextCNN model. The high-dimension original figure can be found in the Supplementary Materials

To visualize our model for sRNA prediction, we draw an overall flowchart for this work, as shown in Fig. 6. The specific details of each process in this flowchart are described in the following sections.

### Select the best length of *k*-mer

Before the construction of our model, the length of *k*-mer should be determined. For a given k, there are two ways of representing the RNA sequence feature. The first is expressed as the frequency of individual k-mer in the sRNA sequence. We take the example of a 3-mer of RNA with four nucleotide bases (A, C, G, and T) at each position, we get, i.e. 64 3-mer features (AAA, AAC, AAA, AAT, ……, TTG, TTT). Then, the sRNA sequence can be represented as a 64-dimensional vector, with each dimension used to record the frequency of a particular 3-mer. After converting the sequences into fixed-length vectors, the *k* was determined by the performance of three traditional machine learning models (including Support Vector Machine (SVM), Random Forest (RF), and Logistic Regression (LR)). We implemented all machine learning models with scikit-learn (version 1.3.0) library of Python. For all three models, we used the default parameters in scikit-learn. The k in these machine learning models was chosen in 2, 3, or 4.

### Test by three sequence-encoded methods

In this study, we use a TextCNN-based model for sRNA identification. Before TextCNN performs the identification task, we apply three sequence coding methods (*k*-mer, TF-IDF, DistilBERT) to convert the RNA sequences into numerical vectors, and then feed them into the same TextCNN model to test the predictive effect of sRNA identification. The TF-IDF, which is abbreviated by Term Frequency-Inverse Document Frequency, is usually applied in document representations in the field of natural language processing (NLP) [26]. Term Frequency (TF) is to calculate the frequency of *k*-mer in sRNA sequences and Inverse Document Frequency (IDF) is to calculate the universal importance of *k*-mer. The IDF for a given *k*-mer is the total number of sRNA sequences divided by the number of sequences in which the *k*-mer occurs. The TF-IDF value is the TF value multiplied by the IDF value. Thus, TF-IDF tends to ignore common *k*-mer and retain important *k*-mer.

We treat each sRNA sequence in the dataset as sentences in the text, and each *k*-mer as a word in that sentence. The sRNA sequences are then fed into the DistilBERT model, which consists of six encoder layers and 768 hidden layers. In DistilBERT, [CLS] and [SEP] tags are first added to each RNA sequence to ensure that the sequence can be properly embedded into the DistilBERT model. This process DistilBERT generates the appropriate token and position embedding for each *k*-mer so that the model can understand the following information in the sequence. The next step is to process these embedding vectors using the encoder layer. The encoder layer also uses distillation to simplify the multi-head and feed-forward neural network sub-layers of DistilBERT to transform the embedding vectors of the input sequences into more compact feature representations in a more lightweight manner.

### Test the performance of TextCNN algorithm

The output of final features from the last encoder layer of the DistilBERT model will be used as input for the TextCNN-GAN model. The model was trained on NVIDIA P100 GPUs, utilizing their 16 GB of graphics memory. TextCNN is a useful deep-learning algorithm for text classification tasks [27]. We augment the features generated by the DistilBERT model using a GAN algorithm before inputting them into the TextCNN [28]. A generator for GAN was used that maps input features to hidden layers, which are then mapped to the final output features via two linear layers and an activation function. Each convolutional layer was followed by a ReLU activation function that allowed the network to better capture complex patterns and features in the text data. TextCNN then performed a maximum pooling layer on ReLU to obtain the most significant features and reduce the dimensionality of the output vector.

Subsequently, to solve the binary classification problem, a fully connected layer was finally used to act as the key classification module responsible for mapping the features to a space with two output nodes. This fully connected layer learned more advanced feature representations during the training process, providing the model with the ability to effectively classify the input data. Throughout the network, this fully connected layer was the decision layer of the model, outputting the corresponding class probabilities to complete the classification prediction of sRNA sequence data.

We use fivefold cross-validation (fivefold CV) on the training set and the value of ACC to adjust the hyperparameters of TextCNN. Many hyperparameters affect the computational results of the model. In this study, we take the number of convolutional kernels, the activation function of the convolutional layer, the pooling window size, and the dropout rate as the main tuning hyperparameters. Optuna was used to optimize the parameters [29]. The hyperparameter details are shown in Table 7. The hyperparameters are optimized by the "Adam Optimizer" to find the parameters with the highest ACC in the validation dataset. The algorithm used an early stopping strategy to select the best-performing parameter.

**Table 7:** The details of hyperparameters in TextCNN

Model	Hyperparameter	Range
TextCNN	Number of convolution kernels	32, 64, 128, 256, 512, 1024
	Activation functions in convolutional layers	None, relu, tanh, sigmoid
	Max pooling size	2, 3, 4, 5
	Rate of dropout layers	1e-6 ~ 1e-4

### Compare with other prediction tools

To further evaluate the performance of sRNAdeep in identifying bacterial sRNAs, we compared sRNAdeep with existing predictors. We selected current prediction tools according to the following criteria: (1) the availability of a web server or a standalone version; (2) good performances in the identification of sRNAs and related genes; and (3) whether the output is an sRNA or a score. Thus, two prediction tools (sRNARanking and PresRAT) fulfill these criteria. The program packages of sRNARanking [6] (retrieved from https://github.com/BioinformaticsLabAtMUN/sRNARanking) and PresRAT [7] (retrieved from http://www.hpppi.iicb.res.in/presrat/Download.html) were provided for comparison.

We download a dataset from PresRAT containing 1174 sRNA and 5869 non-sRNA sequences. We also downloaded a dataset from sRNARanking containing 163 positive instances and 489 negative instances. Based on this dataset, we retrained sRNAdeep and tested the performance in these two datasets. Four indexes, i.e. sensitivity (SEN), specificity (SPE), accuracy (ACC), and area under the curve (AUC), were applied to evaluate the performance of these three models.

### Evaluation indexes in this study

To evaluate the classification performance of the sRNAdeep predictor, we chose to compute seven metrics: SEN, SPE, ACC, AUC, Matthew’s correlation coefficient (MCC), precision (PRE), and F1-score (FSC). The seven metrics are defined as shown in Eq. (1):

1 $$\left\{\begin{array}{c}SEN=\frac{TP}{TP+FN}\\ SPE=\frac{TN}{TN+FP}\\ ACC=\frac{TP+TN}{TP+TN+FP+FN}\\ MCC=\frac{\left(TP\times TN\right)-(FP\times FN)}{\sqrt{(TP+FP)(TP+FN)(TN+FP)(TN+FN)}}\\ PRE=\frac{TP}{TP+FP}\\ FSC=\frac{2\times SEN\times PRE}{SEN+PRE}\\ AUC:Area under the ROC Curve\end{array}\right.$$

Where TP and TN denote the number of sRNAs and non-sRNAs correctly identified by the predictor, respectively, and FP and FN specifically denote the number of sRNAs and non-sRNAs that cannot be correctly identified by the predictor. AUC refers to the receiver operating characteristic curve.

### Identify sRNAs in MTB

*Mycobacterium tuberculosis* (MTB) is one of the severe health challenges globally with high drug-resistance rates. In our previous studies, we have annotated protein function and identified single nucleotide variants related to the drug resistance mechanism of MTB [14, 30]. Currently, the sRNA of MTB remains unknown. The known sRNAs were aligned against non-coding region of MTB genome by BLAST to identify possible similar RNA sequences. Subsequently, our sRNAdeep was employed to discriminate if these sequences belong to sRNA.

### Analysis target genes regulated by sRNAs

Identifying target genes regulated by sRNA is crucial for the treatment of tuberculosis. In this study, an effective tool, TargetRNA3, was applied to predict targets of sRNA [31]. The protein–protein interaction (PPI) network for sRNA-regulated genes was retrieved from STRING database [32]. The topological characteristics of the network nodes are analyzed by the Cytoscape software [33]. Gene ontology (GO) function enrichment and pathway analysis of these genes were performed by DAVID website [34].

## Supplementary Information

Supplementary Material 1.

Supplementary Material 2.

## Data Availability

Our tool and benchmark dataset can be freely available from https://github.com/pyajagod/sRNAdeep.git

## Abbreviations

ACC: Accuracy
AUC: Area Under the Curve
BERT: Bidirectional Encoder Representations from Transformers
CNN: Convolutional Neural Network
FSC: F1-score
GAN: Generative Adversarial Network
GO: Gene ontology
LR: Logistic Regression
MCC: Matthew’s correlation coefficient
NLP: Natural language processing
RF: Random Forest
SEN: Sensitivity
SPE: Specificity
PRE: Precision
sRNA: Small regulatory RNA
SVM: Support Vector Machine
TF-IDF: Term Frequency-Inverse Document Frequency
