# Supplementary figures and images for: sRNAdeep: a novel tool for bacterial sRNA prediction based on DistilBERT encoding mode and deep learning algorithms
Source: BMC Genomics. 2024 Oct 31;25:1021. doi: 10.1186/s12864-024-10951-6 (PMC11526673; doi:10.1186/s12864-024-10951-6)

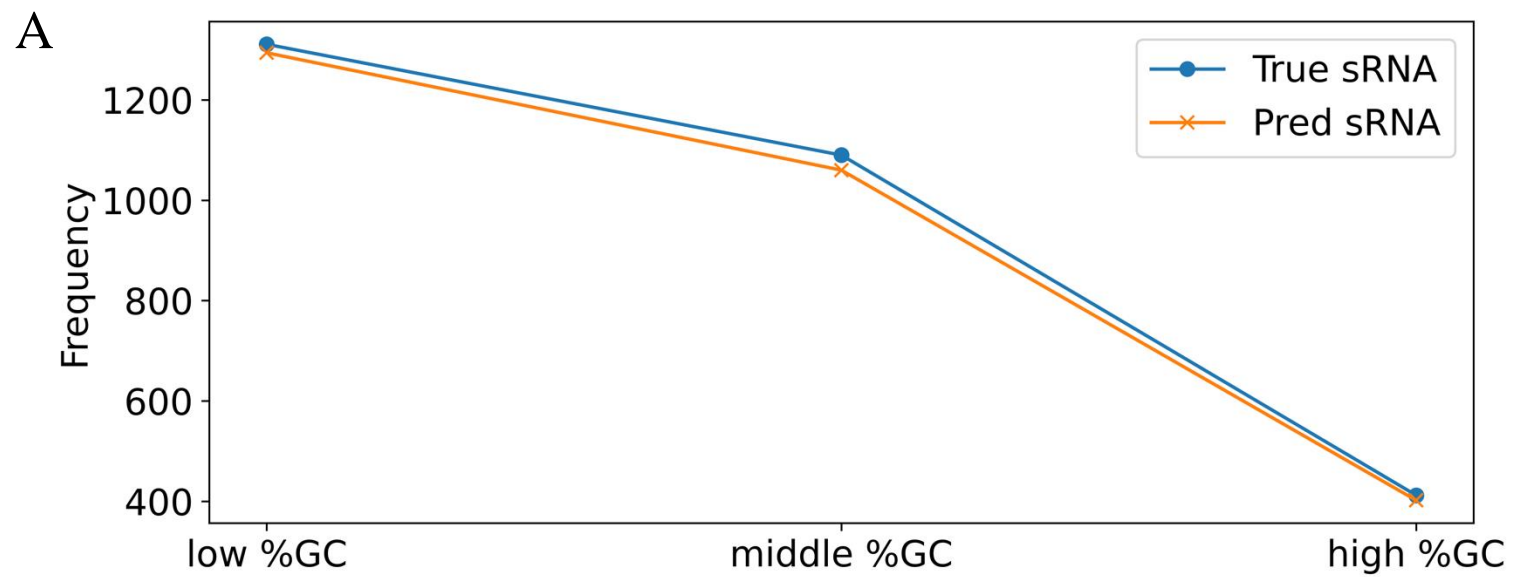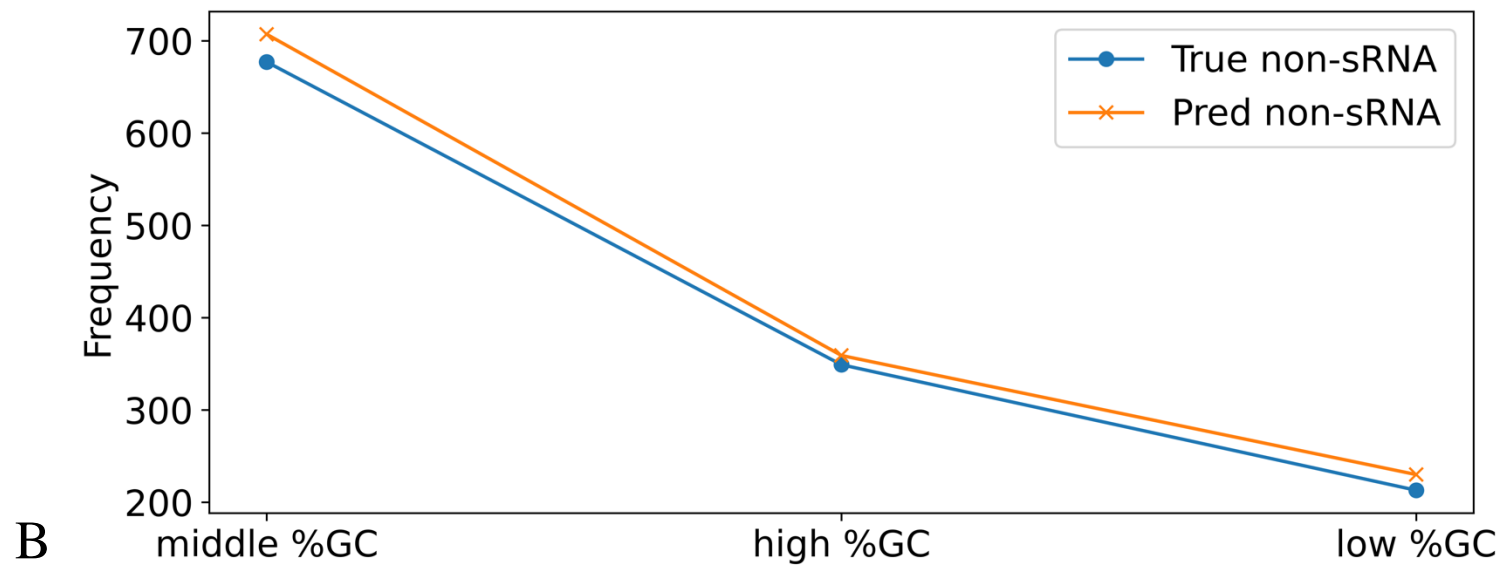

Supplement: Supplementary file 1 — Supplementary Material 1. [file 12864_2024_10951_MOESM1_ESM.pdf]
